# Learning HL7 FHIR Using the HAPI FHIR Server and Its Use in Medical Imaging with the SIIM Dataset

**DOI:** 10.1007/s10278-018-0090-y

**Published:** 2018-05-03

**Authors:** Mohannad A. Hussain, Steve G. Langer, Marc Kohli

**Affiliations:** 1 Agfa Healthcare, Waterloo, ON USA; 2 Imaging Physics and Informatics, Mayo Clinic, Rochester, MN USA; 3 UCSF Medical Center, San Francisco, CA USA

**Keywords:** HL7, FHIR, Health Level 7, Fast Health Interoperability Resources, API, Web APIs, Web-based technology, HAPI, SIIM Hackathon, FOSS, EHR (electronic health record), EPR (electronic patient record)

## Abstract

Health Level 7’s (HL7’s) new standard, FHIR (Fast Health Interoperability Resources), is setting healthcare information technology and medical imaging specifically ablaze with excitement. This paper aims to describe the protocol’s advantages in some detail and explore an easy path for those unfamiliar with FHIR to begin learning the standard using free, open-source tools, namely the HL7 application programming interface (HAPI) FHIR server and the SIIM Hackathon Dataset.

## Introduction to FHIR

**Fig. 1:**
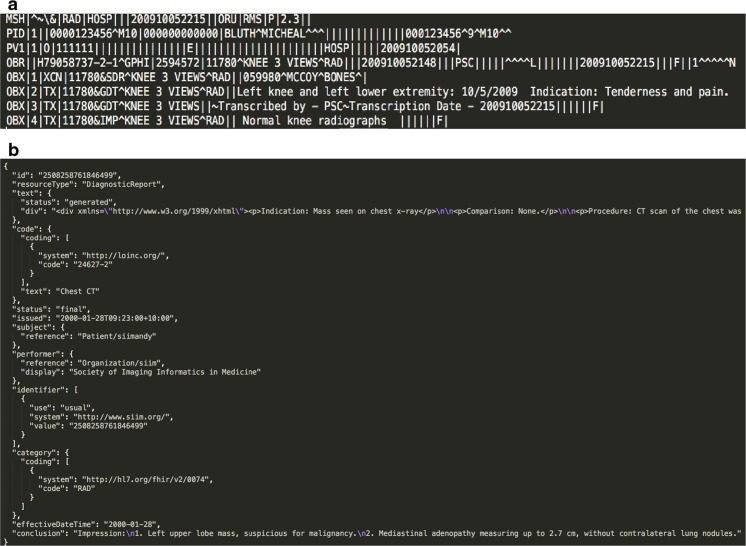
Format comparison between reports in HL7 2.x (**a**) and HL7 FHIR (**b**)

Fast Health Interoperability Resources (FHIR, pronounced “fire”) can be thought of as HL7 (Health Level 7) version 4.0, but that is where any resemblance to other HL7 standards ends [1, 2]. First, FHIR is a departure in design and architecture from both the Arden syntax of version 2, and the SOAP/XML (Service-Oriented Application Protocol, eXtensible Markup Language) of version 3 [3]. Version 2 is commonly employed around the world for point-to-point integration of healthcare systems within a medical center’s firewall, while Version 3 is largely limited to IHE-XDS (a protocol used for data exchange between medical centers) deployments [4]. FHIR was re-designed from the ground up, from data model to network transactions. Rather than using point-to-point TCP/IP connections, FHIR uses the same client-server topology and protocols employed in the World Wide Web (e.g., HyperText Transfer Protocol (HTTP) and RESTful methodology) [5, 6]. Furthermore, unlike previous HL7 standards, FHIR uses a Creative Commons license which makes using it more attractive to the vendor community since licensing fees are not required [7] (see Fig. 1 for a visual comparison between HL7 v2 and FHIR message formats).

Using a RESTful API eliminates the need to choose Windows or Linux line endings, or how to properly format string representations when using unusual delimiters like the pipe character (|), let alone SOAP envelopes. FHIR lowers barriers to implementation, especially for developers unfamiliar with legacy healthcare application protocols (e.g., HL7 2.x), due to adherence to RESTful design principles, which are ubiquitous in modern software development. This ubiquity enables high-quality REST libraries for nearly every programming language.

As with most RESTful APIs, FHIR supports XML and JSON (JavaScript Object Notations) for objects. That means FHIR APIs are not only easier to implement in server-to-server communications, but also underpin many mobile and client-side browser applications. The lightweight nature of JSON in particular makes it easier for applications with limited processing power and/or memory (such as mobile phones) to perform well vs. heaver protocols. This is seen in the rise of a RESTful replacement for IHE-XDS (based on SOAP/XML) with IHE-MHD (Mobile access to Healthcare Documents) integration profile [8].

While FHIR does not replace the ubiquitous HL7 v2 yet, and probably will not for several years, many organizations have already seen the value of adopting FHIR side-by-side with legacy HL7 standards. In addition, there are efforts to “wrap” legacy HL7 information systems with FHIR brokers [9]. Also, existing vendors are beginning to include FHIR with their software upgrades or risk losing customers. In the next section, we describe how to get started with installing an open-source FHIR server and developing applications for it.

## HAPI FHIR Server

HL7 application programming interface (HAPI, pronounced “happy”) has long existed as a go-to library for incorporation of HL7 v2 into applications written in Java [10]. The HAPI community has focused on creation of a FHIR library allowing for both consuming and exposing FHIR APIs. The project is feature rich including: the server, excellent documentation, a supportive community, and frequent releases (fixing bugs, adding features, and expanding support for newer FHIR resources and standards). Additionally, the HAPI FHIR library is compatible with Java 6 and newer versions, which allows it to be easily integrated into older applications. For reference, Java 6 first came out in 2006 and Java is currently on version 9.

One great example of an open-source project that was built on top of HAPI FHIR is the “FHIR Broker” developed jointly by the NIH Sync for Science program and the RSNA Image Share Network, which accepts RESTful FHIR calls and brokers them into existing PACS using traditional DICOM [11].

The HAPI FHIR library is primarily supported by the University Health Network (UHN), a large multi-site network of teaching hospitals in Toronto, Ontario, Canada [12]. The library is open-source with a very generous Apache license v2 [13], allowing the user freedom to use the software for purpose as well as distributing modified versions of the software without concern for royalties.

Although this paper primarily describes setting up a FHIR HAPI server (required to host custom datasets and retain them over a long period of time), it is worth noting that UHN offers a public test server [14]. That site can be useful for a quick reference or a first look at FHIR if you have not used it before.

As the HAPI team likes to emphasize, the HAPI FHIR library was built to be flexible above all else. Some examples of usage scenarios as outlined by the HAPI team include the following:

- Use the HAPI FHIR parser and encoder to convert between FHIR and your application’s data model
- Use the HAPI FHIR client in an application to fetch from or store resources to an external server
- Use the HAPI FHIR server in an application to allow external applications to access or modify your application’s data
- Use the HAPI JPA/Database Server to deploy a fully functional FHIR server you can develop applications against

## SIIM Hackathon Dataset

When SIIM held its inaugural hackathon in 2014 to showcase innovation through new standards like HL7 FHIR and DICOMweb, it became quickly obvious that one of the biggest adoption barriers for these new standards was the lack of a cohesive, rich test dataset that allows developers to build and test applications with data that resembles the real world. In fact, such a dataset would help accelerate the pace of innovation in imaging informatics, a boon for the entire industry. To address this, the SIIM Hackathon committee set out to build a set of five patient narratives, marrying FHIR resources to DICOM images [15]. The narratives are fictitious but believable, along with DICOM images that corroborate each patient’s story. Real-life radiologists heavily contributed to this dataset. The set was created for the first version of the FHIR standard and has been incrementally updated to be compatible with the second and third (most current, also known as STU3) version [16].

The SIIM Hackathon Dataset is distributed under a MIT license and can be loaded onto your own FHIR and/or DICOMweb servers, or used on SIIM’s Hackathon cloud servers (free of charge).

## Building Your Own FHIR Playground

### Prerequisites

#### Java Development Kit

An ordinary Java Runtime Edition (JRE) is not sufficient for our development purposes. We require the Java Development Kit (JDK) to be installed to compile java code, which we need to do to build the FHIR server.

There are two options for a JDK, one provided by OpenJDK (http://openjdk.java.net/) and one provided by Oracle (http://www.oracle.com/technetwork/java/javase/downloads/index.html).

Depending on your operating system, installing the JDK might be done via the package manager/application store (e.g., Ubuntu Linux), or an installer must be downloaded from the JDK provider’s website, as is the case with Windows. Whichever route you take to get your JDK installed, we recommend installing version 8 or newer for the most up-to-date security patches.

#### Java Application Server

We also need a Java application server. Examples include Apache Tomcat, JBoss/Wildfly, Websphere. For this paper, we will focus on Tomcat, specifically version 8. If using Windows, you can easily install Tomcat 8 via the “Windows Service Installer” from https://tomcat.apache.org/download-80.cgi, which conveniently adds Tomcat 8 as a system service, making it easy to start and stop the application server via the Control Panel’s Administrative Tools.

For other platforms such as Ubuntu Linux, an installer might be available via the operating system’s package manager (or application store). If not, binary packages can be obtained from https://tomcat.apache.org/download-80.cgi and Tomcat’s website (or a quick Google search) should point out the exact installation steps for your platform of choice.

**Fig. 2:**
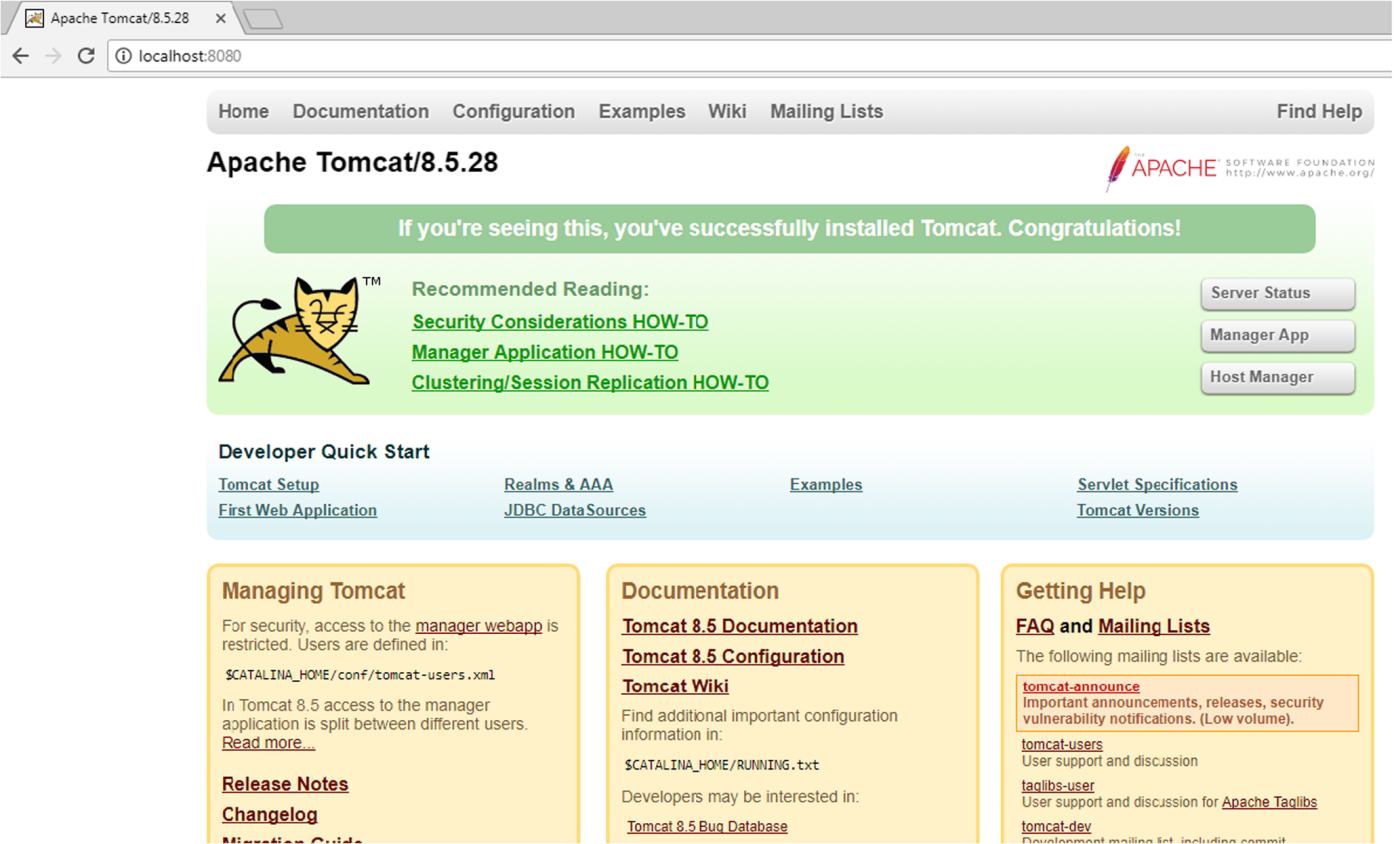
The default index page of a newly installed Apache Tomcat server

Once installed, configured, and fully running, you should be able to see Tomcat’s default index page at http://localhost:8080 (substitute localhost for a hostname or IP address if you are installing it on a remote machine), as seen in Fig. 2.

#### Apache Maven (Java Package Manager)

Apache Maven is required to be able to resolve HAPI’s dependency and compile a complete package that can be deployed onto a Tomcat web server. Luckily, Maven is the easiest component in this puzzle. It can be found at https://maven.apache.org/download.cgi.

**Fig. 3:**
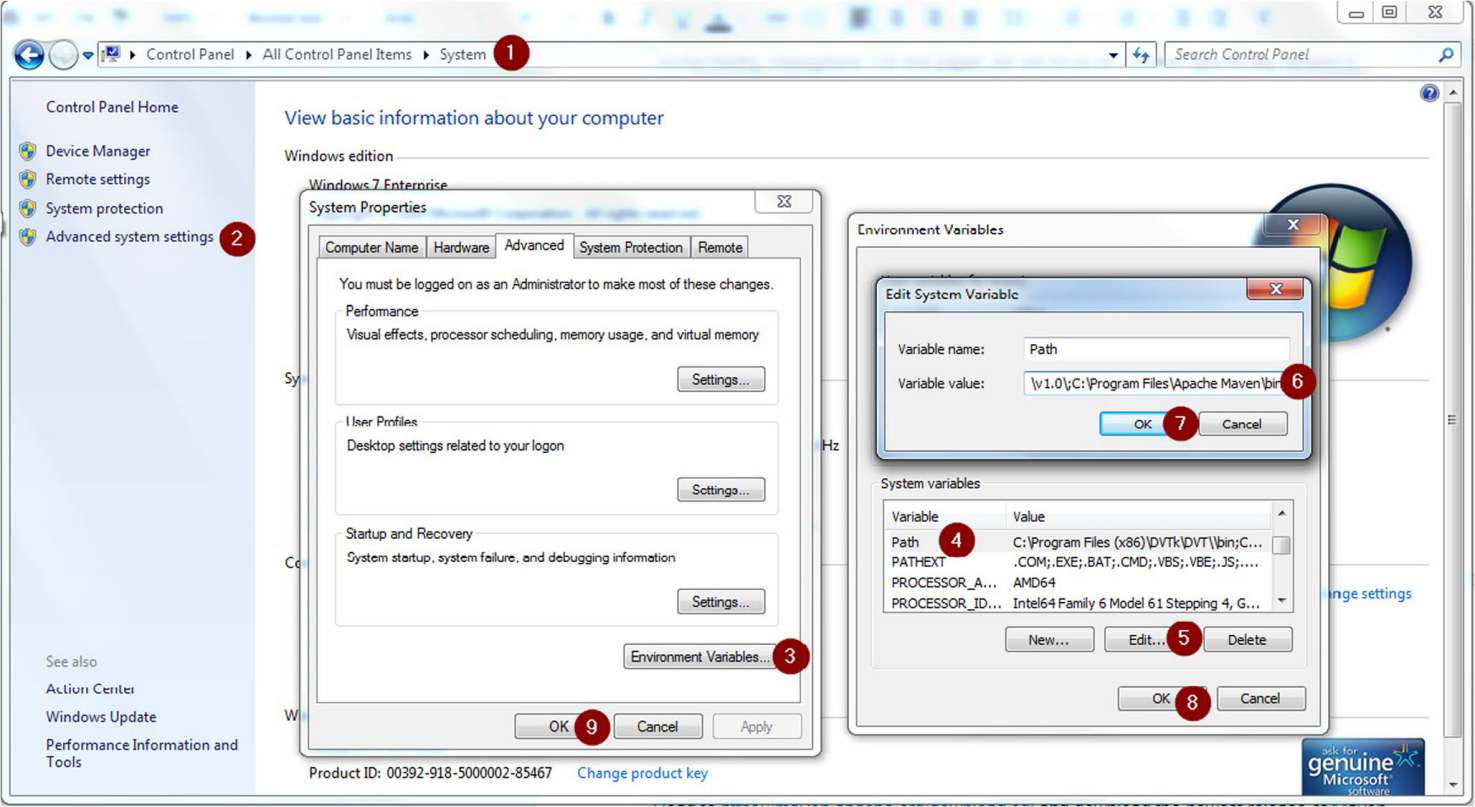
Steps to add Apache Maven into the PATH environment variable under Microsoft Windows

Download the newest release of Maven. Unzip the archive you downloaded anywhere to your file system, then ensure you add the “bin” directory (absolute path) to your operating system’s PATH environment variable. For example, under Windows, one could unzip Maven into “C:\Program Files\Apache_Maven” to keep the default. Then, one adds “C:\Program Files\Apache Maven\bin” to the PATH environment variable as seen in Fig. 3.

#### Ruby and Bundler

One also needs the Ruby programming language and Bundler (a package manager for Ruby) to execute the scripts for loading the SIIM Hackathon Dataset onto your FHIR server. Similar to the above, installation packages might be available via your Operating System’s package manager/application store. If not (e.g., in the case of Windows), Ruby can be obtained from https://www.ruby-lang.org/en/.

Once Ruby has been installed, Bundler can be installed simply by opening a command line window and issuing the command below:

gem install bundler

(see http://bundler.io/ for more information.)

## Compiling and Installing HAPI FHIR

At this point, we are ready to install HAPI from https://github.com/jamesagnew/hapi-fhir/releases. Find the newest ZIP file release of the so-called JPA server (for example, hapi-fhir-jpaserver-example.zip), then unzip somewhere within your file system, e.g., “C:\Projects\hapi-fhir-server”.

Once the archive has been unpacked, open up a command line window and change directory to where the source code resides. Then, run “mvn install” to compile the source code. It may take a few minutes as it downloads all the dependencies and packages everything together. Here is an example of what these commands would look like under Windows:

cd C:\Projects\hapi-fhir-servermvn install

The subdirectory “target” is created (“C:\Projects\hapi-fhir-server\target” if you are following the example convention here). In it, there will be a .WAR file (e.g., hapi-fhir-jpaserver-example.war), which must be deployed to the Tomcat server; there are two options:

- If Tomcat was installed with the option for “manager webapp,” just follow the link from Tomcat’s default index page, then use the “WAR file to deploy” section to upload the WAR file and let Tomcat process it automatically.

OR

(b) Find Tomcat’s “webapps” folder (e.g., C:\Program Files\Apache Software Foundation\Tomcat 8.5\webapps) and copy the WAR file to it. If on Unix/Linux, file permissions may need adjustment so Tomcat can read it.

Now, use the .WAR file’s name (minus the.WAR extension) to form the URL to check that HAPI FHIR was installed correctly via the browser. Keep in mind it may take a few minutes for it to be loaded. For example:

http://localhost:8080/hapi-fhir-jpaserver-example/

Bear in mind the actual URL depends on the following:

- The hostname/IP address of the server you installed Tomcat on
- What port you configured Tomcat to run on
- The exact name of the.WAR file (e.g., the HAPI version, in the example above, it is 3.2.0)

**Fig. 4:**
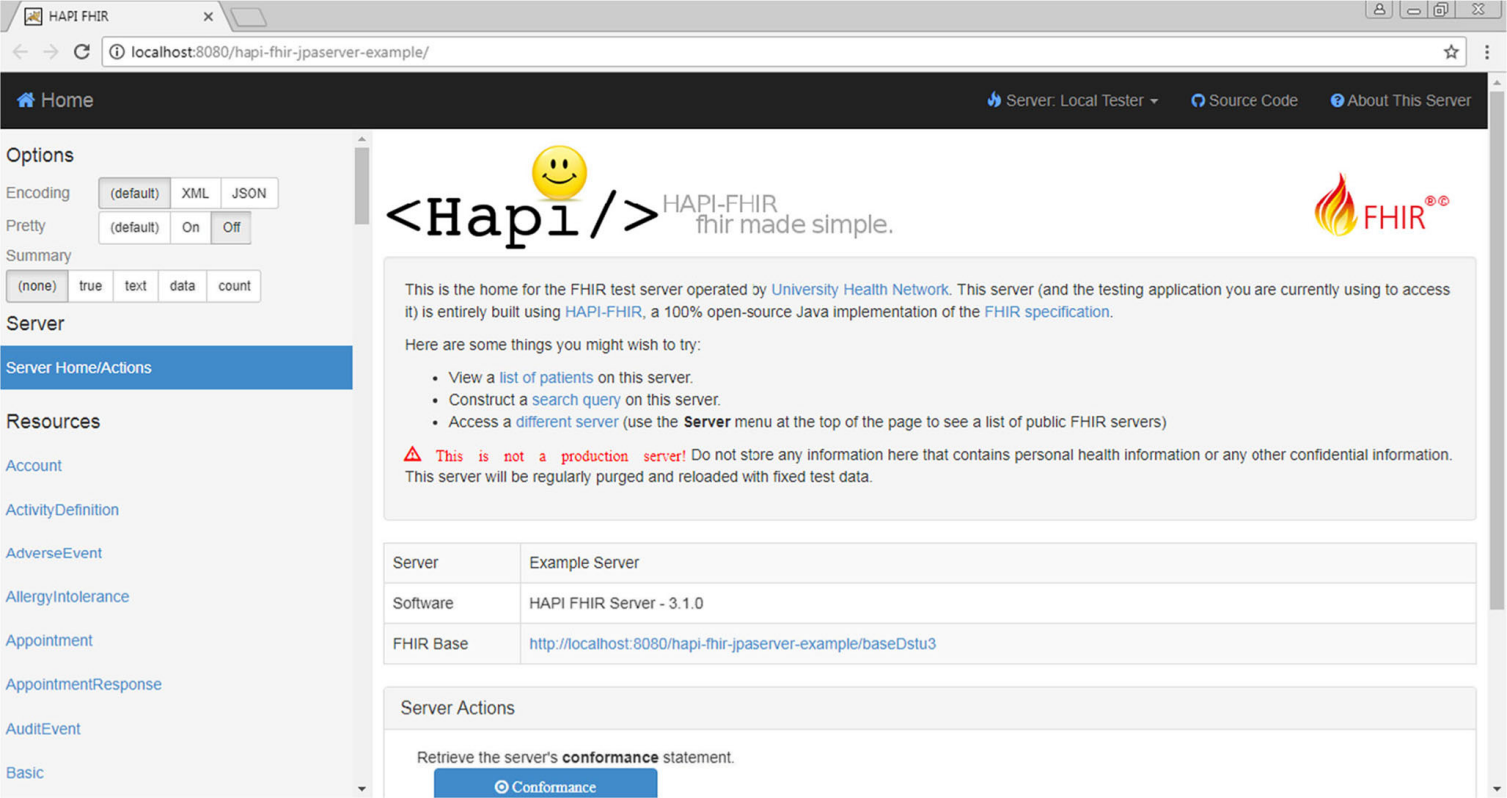
The default index page of the HAPI FHIR server

We should now see a web page similar to Fig. 4.

## Loading the SIIM Hackathon Dataset

Once HAPI is running, you may want to download and use the SIIM Hackathon Dataset. The cleanest way to do that is to clone the SIIM github repository. If you have used Git before, you can simply clone https://github.com/ImagingInformatics/hackathon-dataset.

**Fig. 5:**
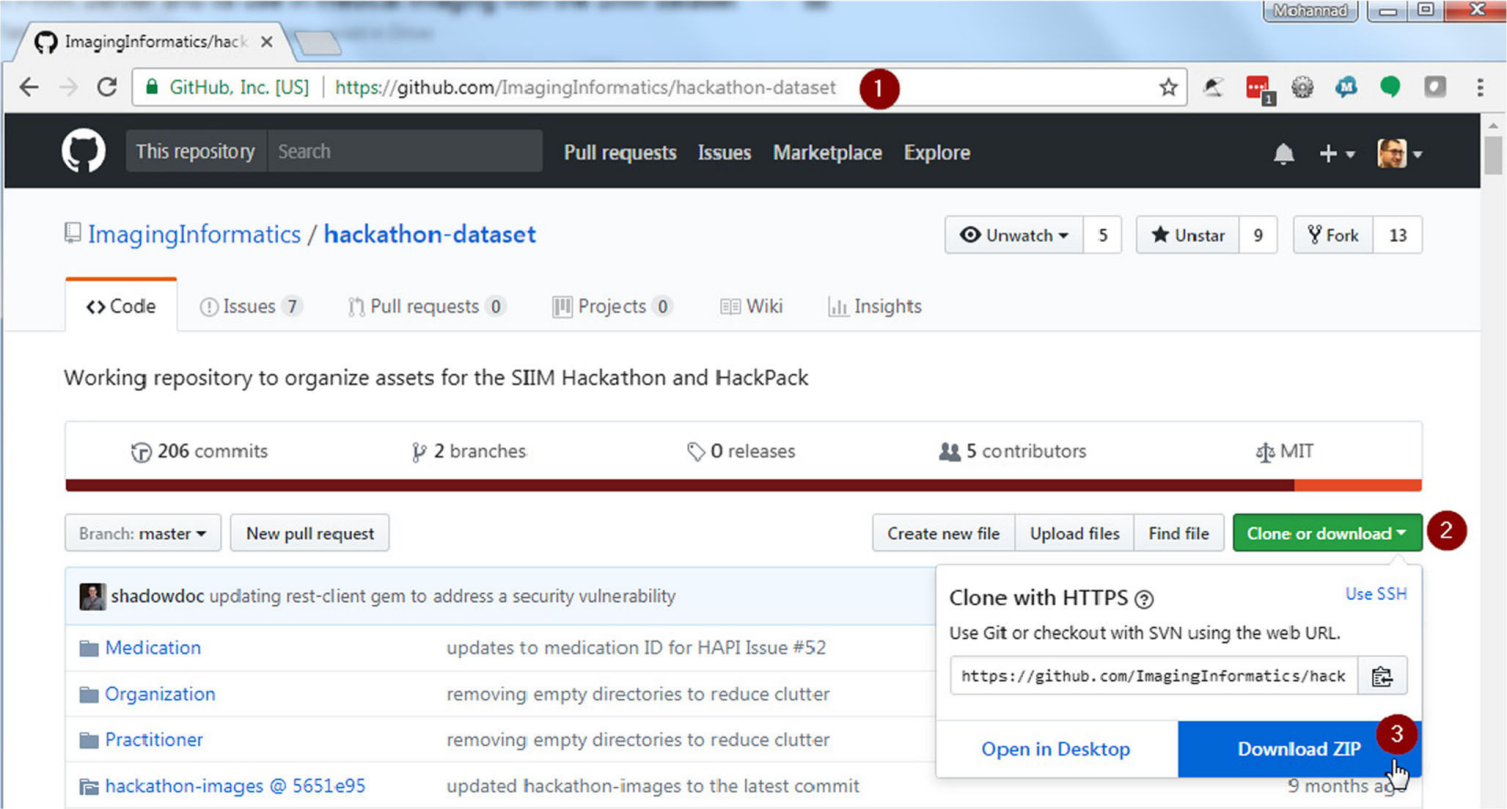
Steps to download the SIIM Hackathon Dataset via github.com

If you have not used git, one can still use the above URL, but select “Clone or download” and “Download ZIP” to download the FHIR dataset (see Fig. 5).

Unzip the folder after it is downloaded. Then, look for the “fhir_server.yml.dist” file and rename it (or create a copy of it) to “fhir_server.yml” (i.e., remove the .dist extension). Then, open the file using a text editor (e.g., Notepad++ in Windows) and update the “url” value to suit your server. Hint. HAPI’s landing page we have seen above provides the full path under “FHIR Base.” Following along the examples above, the URL should look like:

http://localhost:8080/hapi-fhir-jpaserver-example/baseDstu3/

Note: Do not forget the trailing slash. The script will not work without it.

Next, in a command line window, change the directory to where the SIIM dataset was downloaded. A one-time initialization of the Ruby script (to download runtime dependencies) is done via the following command:

bundle install

We are now ready to upload the dataset into the FHIR server, type the following command:

ruby upload.rb fhir_server.yml.

**Fig. 6:**
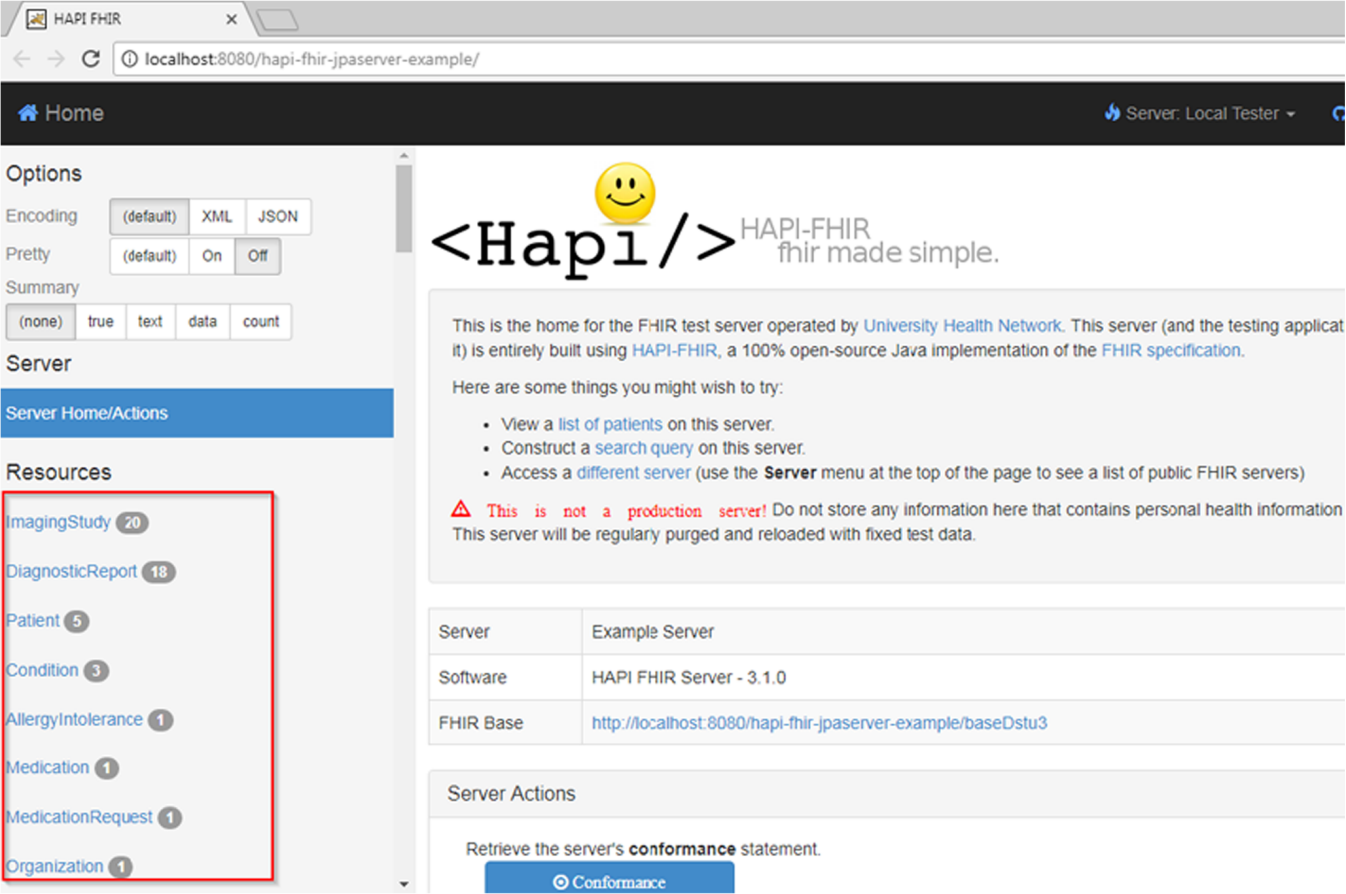
Once the SIIM Hackathon Dataset has been loaded onto the HAPI FHIR server, HAPI’s homepage will show numeric counts next to each object/resource type in the system

Once the script has completed, return to the browser window where we saw the HAPI server’s landing page, select “Refresh” and you should see the numbers next to the FHIR resource names increase, similar to the following screenshot:

Now, one can start developing with the FHIR resources, either using the HAPI UI as seen in Fig. 6, or using tools for testing RESTful APIs, such as Postman [17].

## Conclusion

Many have questioned whether FHIR will move beyond hyperbole, and point to the current “designated for trial use” as an indicator of immaturity. Over the past 3 years, FHIR has seen substantial development, and has graduated out of draft status. Several major electronic health record vendors have at least a partial FHIR implementation, and this has been met with great excitement in the medical imaging and general health IT communities. We believe FHIR delivers tangible improvements at many levels—from application development, to inter-application integration, to easing the entry of developers from other domains (e.g., financial services) as they enter the health IT domain for the first time.

Getting started with FHIR by piecing together a HAPI sandbox server is a relatively easy endeavor, thanks to the availability and ease of installation of open-source packages such as the ones outlined in this paper. It is hoped that this work will be helpful for Certified Imaging Informatics Professionals (CIIPs) and other imaging informaticists learn these new developments in imaging informatics specifically, and health IT in general.
